# Efficacy of Dietary Interventions in Managing Irritable Bowel Syndrome: A Systematic Review of Clinical Outcomes and Gut Microbiota Modulation

**DOI:** 10.7759/cureus.90889

**Published:** 2025-08-24

**Authors:** Hassan Ghega, Zulfiqar Ali, Saad Sohail, Shivam Singla, Bhavna Singla, Zeeshan Kamal, Sunita Kumawat, Simran Kumari, Komal Sohail, Nabila N Anika, Muhammad Usman Fareed

**Affiliations:** 1 Internal Medicine, District Headquarters Teaching Hospital, Gujranwala, PAK; 2 Internal Medicine, Chandka Medical College, Larkana, PAK; 3 Internal Medicine, Mayo Hospital, Lahore, PAK; 4 Internal Medicine, TidalHealth Peninsula Regional, Salisbury, USA; 5 Internal Medicine, Erie County Medical Center (ECMC) Hospital, Buffalo, USA; 6 Internal Medicine, King Edward Medical University, Lahore, PAK; 7 Internal Medicine, Hackensack Meridian Ocean University Medical Center, Brick, USA; 8 Internal Medicine, Sahiwal Medical College, Sahiwal, PAK; 9 General Surgery, University of South Florida Health, Florida, USA; 10 Medicine and Surgery, Holy Family Red Crescent Medical College and Hospital, Dhaka, BGD; 11 General Surgery, Nishtar Medical University, Multan, PAK

**Keywords:** dietary interventions, gut microbiota, irritable bowel syndrome, low fodmap diet, personalized nutrition, quality of life, systematic review

## Abstract

This systematic review explores the effectiveness of dietary interventions in the management of irritable bowel syndrome (IBS), focusing on clinical outcomes and gut microbiota modulation. A broad search of medical literature identified several high-quality studies examining dietary approaches such as low fermentable oligosaccharides, disaccharides, monosaccharides, and polyols (FODMAP) diets, gluten-free regimens, and fiber supplementation. Across the included studies, these interventions were consistently associated with reductions in symptom severity and improvements in quality of life (QOL). In addition, several dietary strategies contributed to favorable changes in gut microbial composition, supporting the role of diet in influencing underlying mechanisms of IBS. Tailored interventions that accounted for individual food sensitivities or triggers demonstrated greater clinical benefit and adherence. Overall, the findings emphasize the potential of personalized dietary modifications as a cornerstone of IBS management. However, further investigation is needed to evaluate long-term outcomes, sustainability, and cost-effectiveness of these approaches across varied patient populations.

## Introduction and background

Irritable bowel syndrome (IBS) is a prevalent functional gastrointestinal disorder marked by recurrent abdominal pain, bloating, and changes in bowel habits, including diarrhea, constipation, or both. It significantly affects patients' quality of life and daily functioning, contributing to substantial healthcare utilization and economic burden worldwide. With an estimated global prevalence of 10-15%, IBS presents a considerable public health challenge due to its complex and multifactorial etiology [1,2]. Factors such as genetic susceptibility, psychosocial stressors, dietary triggers, altered gut motility, and gut-brain axis dysfunction all contribute to its pathophysiology [2]. Among these, growing attention has been directed toward the role of gut microbiota dysbiosis in symptom development and disease progression.

Dietary management is widely regarded as a first-line therapeutic approach for IBS. Multiple studies have shown that nutritional interventions, particularly the low fermentable oligosaccharides, disaccharides, monosaccharides, and polyols (FODMAP) diet, gluten-free diets, and targeted fiber supplementation, alleviate symptoms such as abdominal pain, bloating, and irregular bowel movements [3]. These interventions aim not only to reduce symptom burden but also to minimize dietary triggers and support gut health. In parallel, increasing evidence suggests that dietary patterns can directly influence gut microbial composition, potentially restoring balance and reducing inflammation associated with IBS [4].

Despite these promising outcomes, the clinical utility of dietary interventions remains complicated by interindividual differences in response, dietary adherence, and long-term sustainability. Efficacy varies across IBS subtypes, such as diarrhea-predominant (IBS-D), constipation-predominant (IBS-C), and mixed-type IBS (IBS-M), making it difficult to recommend a one-size-fits-all dietary plan [5]. Furthermore, while the modulation of gut microbiota is recognized as a potential mechanism of action, the exact relationship between dietary changes, microbial shifts, and symptom improvement remains to be fully elucidated [6].

Given the rising clinical interest and the need for personalized treatment strategies, this systematic review aims to evaluate the efficacy of dietary interventions in managing IBS symptoms and their influence on gut microbiota composition. By synthesizing current evidence across various dietary approaches, the objective of this review is to inform clinical decision-making and identify key areas for future research in the dietary management of IBS.

## Review

Materials and methods

Search Strategy

The search strategy for this systematic review was developed using the Patient, Intervention, Comparison, Outcome (PICO) framework [7]. A comprehensive search of PubMed, Medical Literature Analysis and Retrieval System Online (MEDLINE), and Embase was conducted for studies published between January 2013 and December 2023, focusing on adults with IBS undergoing dietary interventions such as low FODMAP diets, gluten-free diets, or fiber supplementation, with outcomes including symptom improvement, gut microbiota changes, and quality of life. Search terms combined Medical Subject Headings (MeSH) and free-text keywords using Boolean operators. For example, the PubMed search string was: ("Irritable Bowel Syndrome"[Mesh] OR "IBS") AND ("Dietary intervention" OR "Diet therapy"[Mesh] OR "low FODMAP" OR "gluten-free diet" OR "fiber supplementation") AND ("Gut microbiota"[Mesh] OR "microbiome") AND (randomized controlled trial[Publication Type] OR clinical trial[Publication Type]). Equivalent strategies were applied in MEDLINE and Embase with corresponding controlled vocabulary. Filters limited results to randomized controlled trials and clinical studies in adults. The process followed the Reporting Items for Systematic Reviews and Meta-Analyses (PRISMA) guidelines [8], with duplicates removed and two reviewers independently screening all records against predefined criteria to ensure methodological rigor and reliability.

Eligibility Criteria

The eligibility criteria [9] for this systematic review were established to ensure the inclusion of high-quality studies that directly addressed the objectives of evaluating dietary interventions for IBS. Studies were included if they focused on adults diagnosed with IBS using established criteria such as Rome III or IV, provided detailed interventions involving dietary modifications (e.g., low FODMAP diet, gluten-free diet, or fiber supplementation), and reported clinically relevant outcomes such as changes in IBS symptom severity (IBS-SSS), quality of life, or gut microbiota composition. Only randomized controlled trials (RCTs) and clinical trials published in peer-reviewed journals were considered, ensuring a robust evidence base. Additionally, studies had to have at least one comparison group, such as traditional dietary advice or pharmacological treatment, to facilitate meaningful comparisons.

Exclusion criteria included studies focusing on pediatric populations, those not providing clear diagnostic criteria for IBS, or interventions not primarily dietary, such as pharmacological or psychological treatments. Studies lacking measurable clinical or microbiological outcomes, case reports, reviews, and non-English publications were also excluded to maintain the review’s methodological rigor. These criteria were designed to balance comprehensiveness with specificity, ensuring that the included studies provided relevant and actionable insights into the effectiveness and mechanisms of dietary interventions for IBS management.

Data Extraction

Data extraction for this systematic review was conducted systematically and meticulously to ensure accuracy and consistency across all included studies. Key details such as study design, population characteristics (e.g., diagnostic criteria, IBS subtype, sample size), intervention protocols (e.g., type, duration, and dietary components), comparison groups, primary and secondary outcomes (e.g., IBS-SSS, quality of life, gut microbiota changes), and key findings were extracted using a predefined data extraction form. Multiple reviewers independently extracted data to minimize bias, with discrepancies resolved through discussion or by consulting a third reviewer. The process adhered to PRISMA guidelines, ensuring transparency and reproducibility. Where necessary, authors of included studies were contacted for clarification or additional data. This structured approach to data extraction provided a comprehensive dataset for synthesis and analysis, forming the foundation for the review’s conclusions and recommendations.

Data Analysis and Synthesis

The data analysis and synthesis process in this systematic review involved a qualitative approach, given the heterogeneity in study designs, interventions, and outcome measures among the included studies. Extracted data were systematically summarized in tables and narratively synthesized to identify patterns, trends, and key findings related to the efficacy of dietary interventions for IBS. Primary outcomes, such as reductions in IBS-SSS, and secondary outcomes, including changes in gut microbiota composition and quality of life, were compared across studies to highlight consistencies and variances. Subgroup analyses were performed when possible, focusing on specific interventions (e.g., low FODMAP vs. gluten-free diet) and IBS subtypes (e.g., IBS-D). Statistical pooling or meta-analysis was not conducted due to the variability in methodologies and reported outcomes, but the narrative synthesis allowed for meaningful integration of findings and identification of overarching themes. This approach ensured a comprehensive understanding of the current evidence base, enabling the formulation of clinically relevant conclusions and recommendations.

Results

Study Selection Process

**Figure 1:**
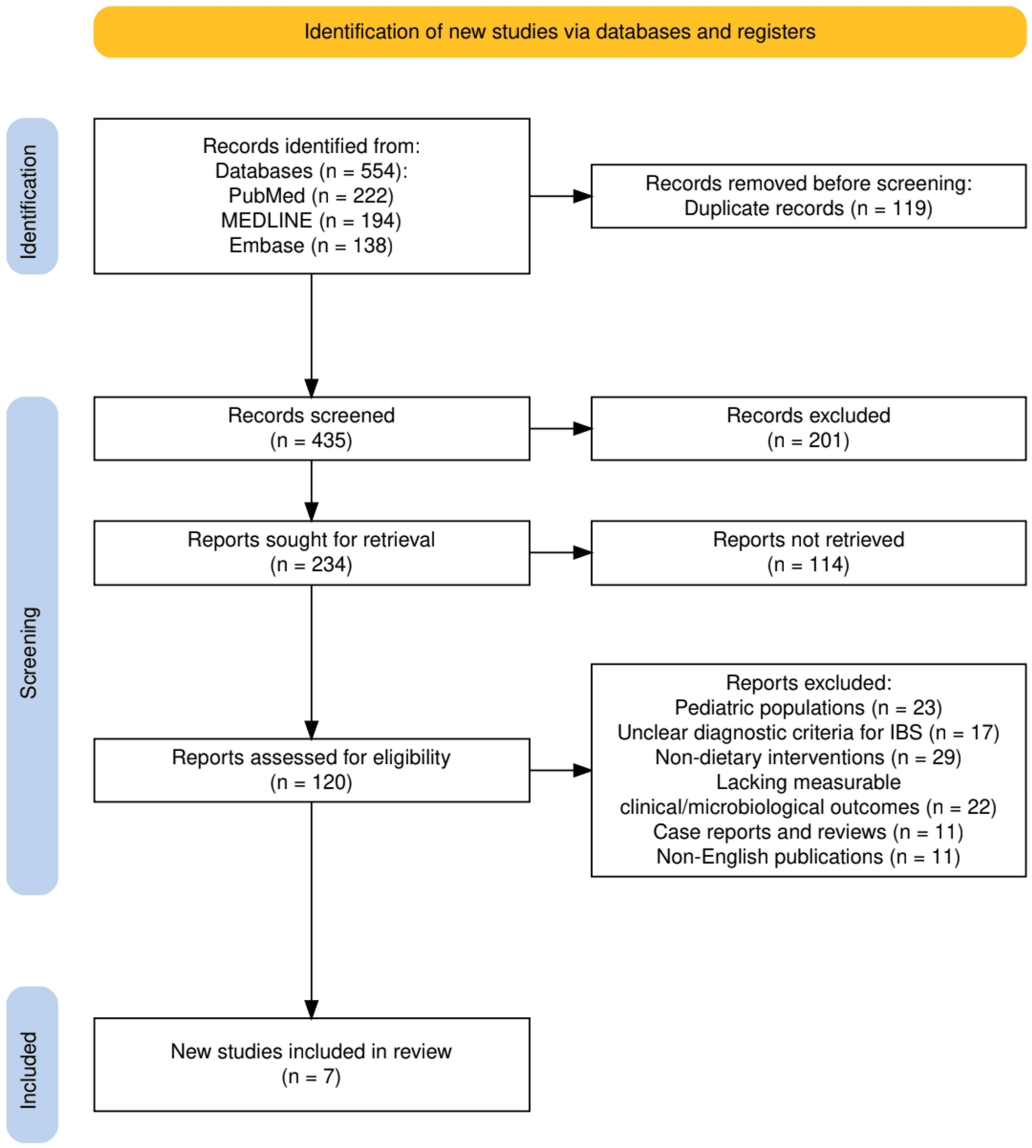
The PRISMA flowchart represents the study selection process PRISMA: Preferred Reporting Items for Systematic reviews and Meta-Analyses

The study selection process for this systematic review followed the PRISMA guidelines, as outlined in Figure 1. A total of 554 records were identified through comprehensive database searches across PubMed, MEDLINE, and Embase. After removing 119 duplicates, 435 unique records were screened based on titles and abstracts, leading to the exclusion of 201 irrelevant studies. The remaining 234 articles were sought for full-text retrieval, of which 114 could not be accessed. Subsequently, 120 full-text articles were assessed for eligibility, and 113 were excluded for reasons such as focusing on pediatric populations, unclear IBS diagnostic criteria, non-dietary interventions, or lacking measurable clinical or microbiological outcomes. Ultimately, seven studies were included in the final review. This rigorous selection process, illustrated in Figure 1, ensures a robust and transparent approach to synthesizing high-quality evidence on dietary interventions for IBS.

Characteristics of the Selected Studies

The characteristics of the selected studies, as summarized in Table 1, highlight the diversity in populations, interventions, and outcomes evaluated in this systematic review. The studies included adults diagnosed with IBS using established criteria such as Rome III or IV, with sample sizes ranging from 42 to 294 participants. Interventions predominantly focused on the low FODMAP diet, either alone or combined with other dietary strategies such as gluten-free diets or fiber supplementation, with durations varying between three and 16 weeks. Comparison groups included traditional dietary advice (TDA) or pharmacological treatments, ensuring robust evaluations of dietary efficacy. Primary outcomes typically centered on reductions in IBS-SSS, as measured by the IBS-SSS, while secondary outcomes explored gut microbiota composition, quality of life, and psychosocial comorbidities. The study designs included RCTs and clinical trials, providing high-quality evidence to evaluate the impact of dietary interventions on IBS management. These characteristics illustrate the comprehensive nature of the review, encompassing a wide range of dietary approaches and their multifaceted impacts on IBS symptoms and underlying mechanisms.

**Table 1:** Characteristics of included studies evaluating dietary interventions in irritable bowel syndrome IBS: irritable bowel syndrome; FODMAP: fermentable oligosaccharides, disaccharides, monosaccharides, and polyols; RCT: randomized controlled trial; GFD: gluten-free diet; LFD: low FODMAP diet; TDA: traditional dietary advice; LF-GFD: low FODMAP and gluten-free diet; QOL: quality of life; NICE: National Institute for Health and Care Excellence; SCFA: short-chain fatty acid; FC: fecal calprotectin

Study ID/Author (Year)	Population (P)	Intervention (I)	Comparison (C)	Outcomes (O)	Study Design	Key Findings
Nybacka et al., 2024 [10]	Adults (n=294) with moderate-to-severe IBS (Rome IV; IBS-SSS ≥175), mean age 38 years (SD 13), 82% female, treated at Sahlgrenska University Hospital, Sweden.	LFTD diet (low FODMAP + traditional IBS dietary advice) for four weeks, with reintroduction guidance during follow-up. Another group received a low-carb, high-protein, high-fat diet.	Optimized medical treatment targeting predominant IBS symptoms (open-label).	Reduction in IBS symptom severity (≥50-point reduction in IBS-SSS); safety (adverse events).	Single-centre, single-blind RCT.	Both diets significantly reduced symptom severity compared to optimized medical treatment (p=0.023). A larger effect size was observed in dietary groups.
Rej et al., 2022 [11]	Adults with Rome IV-defined non-constipated IBS (n=99), randomized into three groups (TDA, LFD, GFD).	Three dietary interventions: traditional dietary advice (TDA), low FODMAP diet (LFD), and gluten-free diet (GFD) for four weeks.	TDA compared to LFD and GFD.	Primary: ≥50-point reduction in IBS-SSS. Secondary: Changes in dysbiosis index, quality of life, and nutritional intake.	Randomized controlled trial (RCT).	All diets were effective (TDA: 42%, LFD: 55%, GFD: 58% responders). TDA was more cost-effective and easier to follow than LFD or GFD. Dysbiosis indices did not significantly differ.
Yan et al., 2020 [12]	Adults with IBS consuming a low FODMAP diet (n=XX; sample size not specified in abstract).	Fibre-fix dietary fiber supplement added to an existing low FODMAP diet for three weeks.	Placebo group maintaining a low FODMAP diet.	Primary: Changes in gut microbiome composition, SCFA levels, stool pH, and gastrointestinal symptoms. Secondary: Sleep, QOL, mental health.	Randomized, double-blind, placebo-controlled trial.	Fibre-fix supplementation may modulate gut microbiota, increase SCFA production, and improve sleep, mental health, and QOL in IBS patients.
Van den Houte et al., 2024 [13]	Patients with IBS (n=117), diagnosed using Rome IV criteria, recruited from tertiary-care clinics.	A six-week low FODMAP elimination diet followed by a nine-week blinded reintroduction phase using FODMAP powders (e.g., fructans, mannitol, lactose).	Control: Glucose powder during the reintroduction phase.	Primary: Symptom recurrence (≥50-point rise in IBS-SSS) to specific FODMAP triggers. Secondary: QOL and psychosocial comorbidities.	Randomized, blinded, reintroduction phase following an elimination diet.	The low FODMAP diet significantly improved IBS symptoms (80% responders). Fructans and mannitol were the most common triggers identified during reintroduction.
Zhang et al., 2021 [14]	Chinese IBS-D patients (n=108) diagnosed using Rome III criteria. Participants were recruited from non-Western dietary backgrounds.	Low FODMAP diet (LFD) for three weeks.	Traditional dietary advice (TDA) based on NICE guidelines for IBS.	Primary: ≥50-point reduction in IBS-SSS. Secondary: Changes in fecal microbiota, SCFA levels, and symptom patterns (e.g., stool frequency, wind).	Parallel-group RCT.	Both diets reduced symptoms, but LFD achieved faster improvements in stool frequency and wind. LFD was associated with microbiota changes and decreased saccharolytic activity.
Naseri et al., 2021 [15]	Iranian patients with IBS (n=42) diagnosed using Rome IV criteria; mean age 37.8 ± 10.7 years.	Low FODMAP and gluten-free diet (LF-GFD) for six weeks.	Baseline measurements for gut microbiota and fecal calprotectin (FC) were compared pre- and post-intervention.	Primary: Reduction in IBS-SSS and FC levels. Secondary: Gut microbiota diversity (e.g., Firmicutes/Bacteroidetes ratio).	Clinical trial.	LF-GFD significantly reduced IBS-SSS (P=0.001) and normalized gut microbiota diversity, decreasing the Firmicutes/Bacteroidetes ratio and FC levels.
Goyal et al., 2021 [16]	Patients with diarrhea-predominant IBS (IBS-D, n=101), diagnosed using Rome IV criteria, mean age of 41.9 ± 17.1 years, and 58% male.	Phase I: Strict low FODMAP diet (LFD) for four weeks. Phase II: Modified LFD with systematic FODMAP reintroduction from four to 16 weeks.	Traditional dietary advice (TDA) for IBS symptoms.	Primary: >50-point reduction in IBS-SSS. Secondary: Changes in IBS-QOL, dietary compliance, and nutrient intake.	Prospective randomized trial.	LFD showed significantly greater symptom and QOL improvements than TDA at both four weeks (62.7% vs. 40.8% responders, P=0.0448) and 16 weeks (52.9% vs. 30.6%, P=0.0274). Compliance declined over time.

Quality Assessment

The quality assessment of the included studies, summarized in Table 2, indicates an overall low risk of bias across most domains, demonstrating the reliability of the evidence synthesized in this review. The studies were generally well-designed RCTs with robust randomization processes, appropriate control groups, and adequate reporting of results. Key strengths included the consistent use of validated outcome measures, such as the IBS-SSS, and comprehensive reporting of intervention protocols. However, some studies exhibited minor concerns, particularly related to missing data, variability in outcome measurement, and long-term compliance rates. For example, studies with smaller sample sizes or variability in reporting (e.g., Yan et al., 2020; Naseri et al., 2021) presented challenges to generalizability. Despite these limitations, the overall methodological rigor of the studies included supports the reliability of the findings and their applicability to clinical practice. This thorough quality assessment ensures that the conclusions drawn from the review are based on high-quality and transparent evidence.

**Table 2:** Risk of bias assessment of included studies IBS: irritable bowel syndrome; RCT: randomized controlled trial; IBS-SSS: irritable bowel syndrome symptom severity score; FC: fecal calprotectin; SCFA: short-chain fatty acid

Study ID	Domain 1: Bias in Randomization	Domain 2: Bias Due to Deviations	Domain 3: Bias Due to Missing Data	Domain 4: Bias in Outcome Measurement	Domain 5: Bias in Selection of Reported Results	Overall Risk of Bias	Comments
Nybacka et al., 2024 [10]	Low Risk	Low Risk	Low Risk	Low Risk	Low Risk	Low Risk	Well-designed RCT with adequate randomization and reporting. No significant biases identified.
Rej et al., 2022 [11]	Low Risk	Low Risk	Low Risk	Some Concerns	Low Risk	Some Concerns	Some variability in the measurement of outcomes; otherwise robust methodology.
Yan et al., 2020 [12]	Low Risk	Low Risk	Some Concerns	Low Risk	Low Risk	Some Concerns	Missing data on sample size introduces uncertainty, but overall results appear reliable.
Van den Houte et al., 2024 [13]	Low Risk	Low Risk	Low Risk	Low Risk	Low Risk	Low Risk	Comprehensive study design with blinded reintroduction phase, minimizing bias.
Zhang et al., 2021 [14]	Low Risk	Low Risk	Low Risk	Some Concerns	Low Risk	Some Concerns	Minor concerns regarding outcome measurement variability and reporting.
Naseri et al., 2021 [15]	Low Risk	Low Risk	Some Concerns	Low Risk	Low Risk	Some Concerns	Limited sample size may affect generalizability; methodology otherwise sound.
Goyal et al., 2021 [16]	Low Risk	Low Risk	Low Risk	Some Concerns	Low Risk	Some Concerns	Decline in compliance rates over time introduces potential bias in longer-term outcomes.

Discussion

This systematic review synthesizes evidence from seven studies evaluating the efficacy of dietary interventions in managing IBS and their associated impacts on symptom severity, quality of life (QOL), and gut microbiota composition. Across the studies, the low FODMAP diet consistently demonstrated significant improvements in IBS symptoms, as measured by the IBS-SSS. For instance, Nybacka et al. [10] reported that both the low FODMAP plus traditional dietary advice (LFTD) diet and the low-carb, high-protein, high-fat diet significantly reduced IBS-SSS compared to optimized medical treatment, with a larger effect size in the dietary groups. Similarly, Zhang et al. [14] highlighted that the low FODMAP diet achieved faster improvements in stool frequency and excessive wind compared to TDA. These findings underscore the effectiveness of low FODMAP and related dietary interventions as a primary management strategy for IBS.

In addition to symptom relief, several studies provided insights into the role of dietary interventions in modulating gut microbiota composition and enhancing overall patient well-being. Naseri et al. [15] observed that a combined low FODMAP and gluten-free diet (LF-GFD) significantly normalized the gut microbiota by decreasing the Firmicutes/Bacteroidetes ratio and reducing fecal calprotectin levels. Yan et al. [12] demonstrated that adding a fiber supplement (fiber-fix) to a low FODMAP diet improved short-chain fatty acid (SCFA) production and enhanced sleep, mental health, and QOL. Furthermore, Van den Houte et al. [13] identified fructans and mannitol as the most common symptom triggers during the reintroduction phase of a low FODMAP diet, emphasizing the importance of individualized dietary strategies. Collectively, these findings highlight the dual benefits of dietary interventions in alleviating IBS symptoms and promoting gut health, paving the way for personalized nutrition approaches in IBS management.

However, feasibility in daily clinical practice remains an important challenge. For instance, Goyal et al. [16] reported a significant decline in dietary compliance over time, with adherence dropping between the initial elimination phase and the longer reintroduction period. This decline reflects the restrictive and resource-intensive nature of diets such as the low FODMAP regimen, which often requires detailed food labeling knowledge, specialized dietetic support, and sustained patient motivation. In real-world settings, such barriers may limit long-term sustainability and reduce effectiveness outside controlled trial environments. Cost and accessibility further complicate adherence, as low-FODMAP or gluten-free foods may not be affordable or widely available in all regions. These factors underscore the need for practical strategies, such as gradual food reintroduction, simplified dietary counseling, and culturally adapted dietary plans, to improve feasibility and patient retention. Incorporating dietitians into multidisciplinary IBS care is particularly valuable, as professional guidance can support adherence, reduce patient burden, and balance dietary restrictions with nutritional adequacy. Thus, while dietary interventions demonstrate clear clinical benefits, their real-world applicability depends heavily on structured implementation and individualized support systems.

The findings of this systematic review reinforce the established role of dietary interventions, particularly the low FODMAP diet, in managing IBS [17]. Consistent with prior studies, our review confirms that the low FODMAP diet significantly reduces symptom severity across various IBS subtypes, with notable improvements in stool frequency, abdominal pain, and QOL [18]. For example, studies like Nybacka et al. [10] and Goyal et al. [16] demonstrated that a low FODMAP diet outperformed TDA and even pharmacological treatments in symptom reduction. These results align with previous literature suggesting that restricting fermentable carbohydrates reduces gas production and intestinal distension, key drivers of IBS symptoms. The review also highlights the potential of personalized approaches, as evidenced by Van den Houte et al. [13], who identified specific FODMAP triggers such as fructans and mannitol during a blinded reintroduction phase. This underscores the need for individualized dietary plans tailored to each patient's tolerance levels.

Beyond symptom relief, the findings extend prior knowledge by shedding light on the mechanisms underlying the observed benefits, particularly in relation to gut microbiota modulation. Naseri et al. [15] and Yan et al. [12] demonstrated that dietary interventions not only improved clinical symptoms but also normalized gut microbial diversity and metabolic activity, evidenced by changes in the Firmicutes/Bacteroidetes ratio and increased short-chain fatty acid (SCFA) production. These results suggest that dietary strategies may exert their effects through restoring gut microbial balance, reducing saccharolytic fermentation, and mitigating intestinal inflammation, as indicated by lower fecal calprotectin levels [19,20]. However, some findings contradict earlier assumptions about the superiority of specific diets; for instance, Rej et al. [11] reported comparable efficacy among TDA, low FODMAP, and gluten-free diets, though TDA was more cost-effective and easier to follow. This highlights the importance of considering practical factors like adherence and accessibility when recommending dietary interventions for IBS [21]. Together, these results emphasize the multifaceted benefits of dietary modifications and provide a foundation for integrating dietary therapy into personalized IBS management strategies.

The findings of this systematic review have significant clinical and practical implications for the management of IBS. The consistent efficacy of the low FODMAP diet, as evidenced across multiple studies, suggests that it should be considered a first-line dietary intervention for patients with moderate-to-severe IBS symptoms, particularly those with diarrhea-predominant IBS [22]. The identification of specific FODMAP triggers, such as fructans and mannitol, highlights the importance of individualized dietary plans, enabling patients to reintroduce tolerable foods while minimizing symptom recurrence. For dietitians, these results underscore the need for structured patient education on implementing and maintaining a low FODMAP or modified dietary approach, with emphasis on the potential benefits of adjunctive strategies such as fiber supplementation to support gut health [23,24]. Physicians should also prioritize dietary counseling as part of a multidisciplinary approach to IBS management, considering the superior patient-reported outcomes and QOL improvements associated with dietary interventions compared to pharmacological treatments [25]. Moreover, given the cost-effectiveness and adherence benefits of TDA, healthcare providers may use it as an alternative or stepping stone for patients unable to commit to stricter regimens, ensuring that dietary strategies remain accessible and tailored to individual needs.

This systematic review boasts several strengths, including a rigorous methodology that involved a comprehensive search strategy across multiple databases to ensure the inclusion of high-quality, peer-reviewed studies. The strict adherence to the PRISMA guidelines for study selection and data extraction enhances the reliability and reproducibility of the findings. By synthesizing evidence from diverse populations and dietary interventions, this review provides a holistic perspective on the efficacy of dietary approaches for IBS management, while the emphasis on gut microbiota modulation as an outcome adds a novel dimension to existing research. However, the review is not without limitations. The included studies exhibited considerable heterogeneity in intervention protocols, study populations, and outcome measures, which may limit the generalizability of the findings. Small sample sizes in some studies and inconsistent reporting of key variables, such as adherence and dietary composition, present additional challenges. Furthermore, language restrictions and the exclusion of unpublished data could introduce publication bias. Despite these limitations, this review offers valuable insights into the role of dietary strategies in IBS, emphasizing the need for standardized methodologies and long-term trials to solidify evidence-based recommendations.

Future research should address several gaps identified in the current evidence base, particularly the long-term impacts of dietary interventions for IBS management. While the short-term efficacy of approaches such as the low FODMAP diet is well-documented, more longitudinal studies are needed to evaluate sustained symptom relief, dietary adherence, and potential nutritional deficiencies associated with restrictive diets. Additionally, there is a need to investigate the cost-effectiveness of these interventions, as affordability and accessibility play a crucial role in patient compliance, particularly in low-resource settings. Future studies should also explore the mechanisms linking dietary changes to gut microbiota alterations and their downstream effects on symptom severity and QOL. Personalized approaches, including the use of microbiome profiling and biomarkers to predict dietary response, hold significant promise but require further validation in large-scale trials. Finally, comparative studies examining different dietary interventions across diverse cultural and dietary backgrounds are essential to provide globally applicable recommendations for IBS management.

## Conclusions

This systematic review highlights the significant role of dietary interventions, particularly the low FODMAP diet, in managing IBS by effectively reducing symptom severity, improving quality of life, and modulating gut microbiota composition. The findings underscore the potential of personalized dietary strategies to address the diverse needs of IBS patients, with evidence supporting the superior efficacy of dietary approaches compared to TDA or pharmacological treatments in certain contexts. By synthesizing data from diverse populations and interventions, this review emphasizes the importance of integrating evidence-based dietary management into clinical practice. However, the variability in outcomes across studies and the need for long-term data call for further research to optimize and tailor these interventions. Overall, this work reinforces dietary therapy as a cornerstone of IBS management and provides a foundation for developing more individualized, effective, and sustainable treatment strategies.
